# Interpersonal autonomic synchrony and sexual satisfaction: A preliminary examination

**DOI:** 10.1192/j.eurpsy.2022.717

**Published:** 2022-09-01

**Authors:** B. Freihart, M. Sears, C. Meston

**Affiliations:** The University of Texas at Austin, Psychology, Austin, United States of America

**Keywords:** physiological synchrony, psychophysiology, Sexual Satisfaction

## Abstract

**Introduction:**

A growing body of literature has increased our understanding of interpersonal autonomic synchrony (IAS), or exchanges across biological systems resulting in physiological covariation. While research suggests that IAS is more likely in relationships characterized by emotional intimacy, no research to date has examined the connection between IAS and the quality of sexual relationships.

**Objectives:**

The current study seeks to elucidate the relationship between IAS and sexual satisfaction (SS) using tasks that have previously been used to assess synchrony (i.e.,gazing, mirroring), as well as several conversation-based tasks (i.e., neutral and sex-related conversation tasks).

**Methods:**

Couples (n=28) completed procedures in a laboratory-based setting where they completed survey measures of SS before connecting to an electrocardiogram. Subsequently, heart rate (HR) data for each dyad were analyzed using a moderated multi-level modeling approach.

**Results:**

IAS was detected, with men reliably predicting the HR of female partners, and women reliably predicting the HR of male partners (respectively, β=0.383, p<0.001; β=0.222, p<0.001). AIC values indicate a better fitting model for men predicting female HR. As such, moderation analyses were conducted for that model by study task, finding a significant interaction between SS and observed IAS during the mirroring task (β=0.004, p=0.009) and neutral-conversation task (β=0.016, p=0.009).

**Conclusions:**

These findings reflect evidence that IAS may be relevant to SS at the couple-level. The ability of couples to coregulate while attempting to synchronize (as in the mirroring task) or exchange information (as in the neutral-conversation task) may meaningfully change how they experience their sexual relationship.

**Disclosure:**

No significant relationships.

